# Use of banker plant system for sustainable management of the most important insect pest in rice fields in China

**DOI:** 10.1038/srep45581

**Published:** 2017-04-03

**Authors:** Xusong Zheng, Yanhui Lu, Pingyang Zhu, Facheng Zhang, Junce Tian, Hongxing Xu, Guihua Chen, Christian Nansen, Zhongxian Lu

**Affiliations:** 1 State Key Laboratory Breeding Base for Zhejiang Sustainable Pest and Disease Control, Institute of Plant Protection and Microbiology, Zhejiang Academy of Agricultural Sciences, Hangzhou 310021, China; 2 Jinhua Plant Protection Station, Jinhua 321017, China; 3 Department of Entomology and Nematology, UC Davis Briggs Hall, Room 367, Davis, CA 95616, USA

## Abstract

To meet the World’s food demand, there is a growing need for sustainable pest management practices. This study describes the results from complementary laboratory and field studies of a “banker plant system” for sustainable management of the rice brown planthopper (BPH) (*Nilaparvata lugens* Stål) – the economically most important rice pest in Asian rice growing areas. The banker plant system consisted of planting a grass species, *Leersia sayanuka*, adjacent to rice fields. *L. sayanuka* is the host plant of a planthopper, *Nilaparvata muiri*. An egg parasitoid, *Anagrus nilaparvatae*, parasitizes eggs of both BPH and *N. muiri*, and its establishment and persistence are improved through plantings of *L. sayanuka* and thereby attraction of *N. muiri*. Laboratory results showed that BPH was unable to complete its life cycle on *L. sayanuka*, and *N. muiri* could not complete its life cycle on rice. Thus, planting *L. sayanuka* did not increase the risk of planthopper damage to rice fields. Field studies showed that BPH densities were significantly lower in rice fields with banker plant system compared to control rice fields without banker plant system.

Rice is the principal food of more than 50% of the world’s population, and for 60% of the Chinese population^1^. It has been estimated that the world’s rice production has to increase drastically over the next three decades to meet the growing food demand in Asia^2^. With expanding cities and declining value of arable land, this increase in rice production cannot be met by expansion of cultivation area but must come from higher productivity from existing agricultural land. Throughout Asia, there has been an intensification of rice production through higher fertilizer and pesticides inputs in the last decades^3^,^4^. Although this intensification has led to yield increases, there is growing concern about the sustainability of current practices^5^. Furthermore, the continent has faced a stark increase in insect pest outbreaks of planthoppers (Homoptera: Delphacidae) and other serious insect pests^5^,^6^ and these pest outbreaks have been directly linked to intensification of rice and reduction of biodiversity in rice-based ecosystem^7^.

The brown planthopper (BPH), *Nilaparvata lugens* (Homoptera: Delphacidae) is one of the key pests in Asian rice production systems, including China^5^,^8^,^9^,^10^. At present, the control of rice pests is highly dependent on frequent applications of broad-spectrum insecticides, and there is widespread concern about their low and inconsistent effects on pest populations and high adverse effects on insect natural enemies of rice pests^5^.

To meet the world’s food demand, there is a growing need for sustainable pest management practices. This means that it requires fundamental changes of current practices. Moreover, it is necessary to adopt rice pest management practices with a markedly reduced reliance on pesticides. A possible solution is “banker plant systems” as part of “ecological engineering”^7^,^11^,^12^. Banker plant systems (as well as ecological engineering systems) involve promotion of plant diversity to enhance pest self-regulatory ecosystem functions, such as, predation and competition, to reduce susceptibility of agricultural crops to native and invasive pests^11^,^13^,^14^. Banker plants may provide resources, such as, shelter, pollen and nectar, or alternative preys^15^,^16^,^17^ to improve the establishment and persistence of beneficial insect populations used to control a specific insect pest. Thus, banker plants are established and managed adjacent to crops, and they may attract insects, which can serve as alternative hosts of arthropod natural enemies when the crop pest populations are low or not available. The first successful banker plant system was developed in 1977 and involved tomato as the banker plant, a parasitoid [*Encarsia formosa* Gahan (Hymenoptera: Aphelinidae)], and a whitefly pest [*Trialeurodes vaporariorum* Westwood (Homoptera: Aleyrodidae)] on tomatoes in greenhouses^18^. The bank plant systems developed for a series of important combinations of crops and pests are present as a Table 1.

In rice systems, banker plant systems have involved planting of sesame (*Sesamum indicum*) as nectar source to promote establishment and persistence of a predatory bug [*Cyrtorhinus lividipennis* (Heteroptera: Miridae)] and of parasitoids [*Anagrus spp.* (Hymenoptera: Mymaridae), *Apanteles ruficrus* (Hymenoptera:Braconidae), *Cotesia chilonis* (Hymenoptera:Braconidae), and *Trichogramma chilonis* (Hymenoptera: Trichogrammatidae)]^19^,^20^,^21^. However, no studies on describing attraction of alternative hosts to parasitoids of rice insect pests were published. *Nilaparvata muiri* is a common planthopper species, which is widely distributed in the rice regions of southern China, Japan, Korea and Vietnam^22^,^23^. The grass species, *Leersia sayanuka*, a rice field weed, is one of the main and a preferred host plant for *N. muiri*^24^.

In this study, we examined critically important elements of the first banker plant system on rice in Asia, in which is a grass species, *L. sayanuka*, is planted adjacent to rice fields to attract a planthopper, *N. muiri*. The hypothesis tested in this study is that *N. muiri* does not attack rice plants, and that is an alternative host for an egg parasitoid, *Anagrus nilaparvatae*, which is the main natural enemy of BPH. As a consequence, we predicted that planting *L. sayanuka* would lead to improved establishment and persistence of *A. nilaparvatae* and therefore enhanced biological control of BPH. Based on laboratory studies, we examined the abilities of: 1) *N. muiri* to complete its life cycle on rice, and 2) BPH to complete its life cycle on *L. sayanuka*. In addition, we studied the functional response by *A. nilaparvatae* to the two planthopper host species (BPH and *N. muiri*). Finally in replicated field study, we quantified rice yields in systems with/without establishment of the banker plant system. The potential of the proposed banker plant system and its complementarity with other ecological engineering approaches were discussed.

## Results

### Parasitism of *A. nilaparvatae* to eggs of both BPH and *N. muiri* derived from different host plants

**Figure 1:**
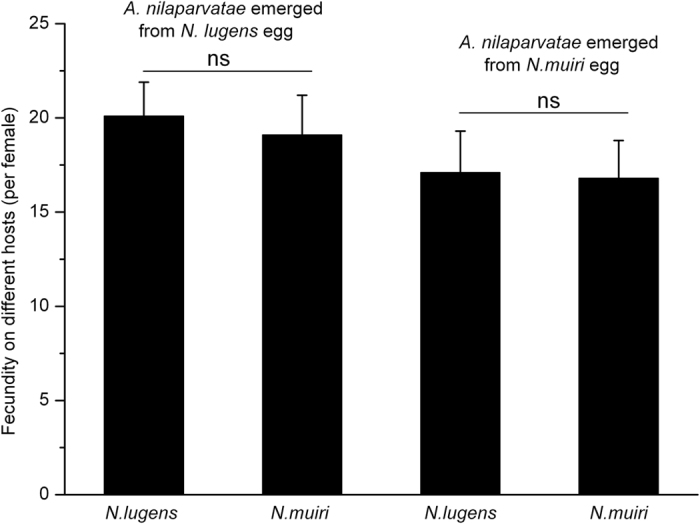
Fecundity of *A. nilaparvatae* from different host plants on different hosts. Ns indicated that there was no significant difference between the *N. lugens* eggs and *N. muiri* eggs treatment by χ^2^ testing (*P* < 0.05).

There were no significant differences in the parasitism of *A. nilaparvatae* to BPH eggs from two host plants (t-test: t = 0.484, df = 58, *p* = 0.876). The oviposition of *A. nilaparvatae* female reared from BPH on BPH and on *N. muiri* eggs were 20.1 and 19.1, respectively. The oviposition of each *A. nilaparvatae* female reared from *N. muiri* on BPH and on *N. muiri* eggs was 17.1 and 16.8, respectively. No significant difference was observed in the ability of *A. nilaparvatae* reared from *N. muiri* to parasitize the two species of planthopper (test: t = 0.404, df = 58, *p* = 0.913) (Fig. 1).

### Functional responses of *A. nilaparvatae* on eggs from two hosts (BPH or *N. muiri*) reared by different host plants

The number of BPH or *N. muiri* eggs parasitized by *A. nilaparvatae* increased with the density of planthopper eggs in the different treatments to an asymptote. Regression analyses of densities of both BPH and *N. muiri* eggs and the parasitized number showed a significant difference among all treatments. The results were consistent with the equation for Holling’s type II functional responses (Table 2). The instantaneous attack rate of *A. nilaparvatae* reared from BPH eggs against BPH was higher than that of *A. nilaparvatae* from *N. muiri* eggs, while the instantaneous attack rates of *A. nilaparvatae* reared from *N. muiri* eggs to BPH and *N. muiri* had no significant difference. The theoretic maximum parasitized eggs numbers of *A. nilaparvatae* reared from the BPH on the two brown planthoppers were not significantly different, while the parasitized egg number of *A. nilaparvatae* from BPH eggs was significantly higher than that of *A. nilaparvatae* from *N. muiri* eggs.

### Oviposition preference of *A. nilaparvatae* reared from different hosts on the eggs of BPH and *N. muiri*

The selectivity index showed that *A. nilaparvatae* derived from hosts had different selectivity toward BPH versus *N. muiri* (Table 3). Between the two planthoppers, *A. nilaparvatae* derived from BPH eggs preferred BPH eggs (χ^2^ = 6.12 > χ^2^_0.05_). With the proportion of BPH eggs to *N. muiri* eggs increased, *A. nilaparvatae*’s preferred *N. lugens* eggs. However, *A. nilaparvatae* derived from *N. muiri* eggs had no obvious preference between BPH eggs and *N. muiri* eggs (χ^2^ = 1.38 < χ^2^_0.05_).

### Field evaluation of the effects of growing *Leersia* planting beside rice plants as a banker plant

The results showed that planting *L. sayanuka* stripes of 10-cm or 50-cm width at the edge of paddy fields kept rice planthopper populations below the threshold density of 15 per hill. The BPH population in the experimental field was significantly lower than that in the control field where the densities of rice planthoppers exceeded the threshold density. There was no significant difference between 10-cm and 50-cm stripes (Table 4). This result suggests that the construction of a 10-cm-wide *L. sayanuka* banker plant system at the edge of a paddy field can achieve effective control of rice planthoppers (mainly BPH).

### Population trend index of BPH and *N. muiri* on different host plants

BPH adults only survived about 2 days on *L. sayanuka* and could not lay eggs. *N. muiri* adults could survive about 5 days on rice plants and could lay only a few eggs (3.6 ± 2.1 eggs per female) and hatching rate was low (23.3%) (Table 5). For the nymphs of *N. muiri*, 32.5% could emerge on rice, which was significantly lower than the emergence rate on *L. sayanuka*. There was no significant difference in the female ratio of *N. muiri* on the two plant hosts. BPH could not complete a generation on *L. sayanuka*, and thus the population trend index was zero. *N. muiri* completed only one generation on rice plants and thus the population trend index was only 0.12, which indicated that *N. muiri* was unable to establish a sustained population. *N. muiri* however had high reproduction ability on *L. sayanuka*, and the population trend index was 44.40% of BPH on rice (Table 4).

## Discussion

As an innovation for classical biological control, the banker plant system is used to sustain a reproducing population of natural enemies in order to provide long-term pest suppression^25^,^26^,^27^. Even in conditions of low pest density natural enemies could maintain sufficient populations on alternative hosts from the banker plants^28^. In addition, this method can be more economical than other classical biological control methods that require more manpower and material resources^25^,^29^. The use of banker plants which are common and easy to cultivate as in the case of *Leersia* can thus be introduced as an ecological engineering method.

Although a few research suggests that rice crops may be the host of *N. muiri*^30^, most researchers have shown that it could not sustain on rice^24^,^31^,^32^. Our results were consistent with this observation as although *N. muiri* could complete a generation on rice crops, their population trend index was only 0.12. Meanwhile, BPH could not survive on *L. sayanuka*, and thus could not serve an alternate breeding source of the pest.

*A. nilaparvatae* populations derived from the eggs of BPH and *N. muiri* had the similar parasitzation capacities for the two brown planthoppers. *A. nilaparvatae* from *N. muiri* eggs had no selection preference between the eggs of BPH and *N. muiri*, suggesting that they do not need an adaptive phase to BPH but can directly play an efficient control role. Meanwhile, *A. nilaparvatae* from paddy fields preferred to parasitize the BPH eggs, indicating that *A. nilaparvatae* rarely moves back to *L. sayanuka*, if there are enough BPH pests in rice fields. *A. nilaparvatae* derived from *N. muiri* had similar instantaneous attack rates and handling times for the two planthoppers’ eggs. The instantaneous attack rates and handling times for BPH were higher suggesting higher efficiency for the control of BPH.

In the paddy fields of China, *N. muiri* populations are generally high. Luo *et al*.^23^ reported that *N. muiri* accounted for over 40% of the total number of *Nilaparvata* insects captured in 2008 and 2009 by using light traps in four provinces and five locations in China. Moreover, the number of *N. muiri* sampled from Jiangxi, Hu’nan, and Zhejiang provinces before September were higher than BPH. Our unpublished data of field experiment also showed that the larger *A. nilaparvatae* populations were often found on *L. sayanuka* (unpublished data)*. L. sayanuka* was introducd into the paddy field ecosystem as a banker plant, which can serve as the reservoir of the parasitic wasps for *A. nilaparvatae* for the management of rice planthoppers seems feasible. As *L. sayanuka* is the perennial graminaceuous plant and it can also serve as an overwintering host plant for parasitoids as well as other predators. Thus sufficiently high populations of *A. nilaparvatae* may be available after winter before rice planthopper immigration occurs. The field experiment showed that a 10-cm-wide *L. sayanuka* strips planted at the edges of paddy fields might be sufficient in suppressing rice planthopper population to below the threshold.

Previous studies have indicated that the spatial pattern of non-crop habitat can affect the composition, structure, diversity and dynamic of natural enemies^33^,^34^. Different species of natural enemies showed different responses to non-crop habitats^35^,^36^, and the number and variety of natural enemies might not be able to improve the biological control ability^37^,^38^. Caballero-López *et al*.^39^ found that the contiguous of crop habitat and habitat diversity have no obvious effect on the number of aphids, parasitoids and beetles, however, the crop area size reduction can enhance carabids. Our study indicated that there was no significant difference between the 50-cm-wide strip and 10-cm-wide strip of *L. sayanuka*. Environmental factors are also important in biological control using a banker plant. For instance, in the “Oat - *Myzus persicae* - *Aphidius colemani*” banker plant system, efficient biological control was achieved in a short period of the winter crops (*Eruca sativa*). However, it was not effective in the longer period of the summer crops (*Capsicum annuum* L.)^40^. In addition, the LNA banker plant system might also be challenged with different rice cultivations, rice varieties and rice field locations. Although the parasitoid is extremely susceptible to early season insecticide applications, it is vitally important when banker plants are used and early season insecticide applications should be avoided.

One risk in using the LNA banker plant system might arise from the creeping stems of *Leersia* that might affect rice as a weed. However, *L. sayanuka* grows more slowly than *Leersia hexandra* Swartz which is an important weed in rice ecosystem. In addition, some rice diseases might affect *L. sayanuka* and might serve as a disease reservoir for instance bacterial blight of rice (*Xanthomonas oryzae* pv. Oryzae)^41^. *Leersia* is also a host for the rice leaf folder, *Cnaphalocrocis medinalis*^42^,^43^ and *Hydrellia philippina* Ferino^44^. Hence, it is necessary to further evaluation the potentials risk of this LNA banker plant system. Spiders and other predators always preyed on *N. muiri*. Our suggested system might notably influence the paddy field ecosystem and nutritional relationships among arthropods. Although *L. sayanuka* may be the host of *C. medinalis* and *Susumia exigua*^43^, the eggs and larvae of these two rice leaf folders in *L. sayanuka* may be the host of natural enemies for the rice lepidopteran pests, and improve the abundance of egg and larva parasitoids, including *Trichogramma japonicun, Trichogramma chilonis*, and *Apanteles cypris*. Thus, *L. sayanuka* strips planted at the edge of paddy fields also benefits to serve as a natural enemy bank of rice lepidopteran pests, such as rice leaf folder.

## Methods

### Plants

A BPH susceptible variety of rice (TN1) was originally obtained from the International Rice Research Institute (IRRI), Philippines. After indoor germination, seedlings were grown in a standard potting mix in a climate controlled room (25 ± 1 °C; 12 h: 12 h = L: D; RH = 80%). Fifteen-day-old rice seedlings were individually transplanted into plastic pots (diameter 10 cm, height 12 cm) and used in laboratory studies.

*L. sayanuka* plants were originally collected from a wetland field on the outskirts of Hangzhou (120°15′E, 30°11′N), and then those plants were transferred to a greenhouse. At the tiller stage, the primarily tillers were cut and transplanted into plastic pots (diameter 10 cm, height 12 cm) and used in laboratory studies.

Both TN1 and *L. Sayanuka* plants kept insects during cultivation free. After 50-days of plant transplanting, each potted rice plant was trimmed to main stem and two primarily tillers and each potted *L. sayanuka* was trimmed to main stem and four primarily tillers in order to maintain equivalent biomass in the experimental pots.

### Insects

Colonies of BPH and *N. muiri* were established from specimens collected from paddy fields at the Jinhua experimental station in Zhejiang Province, China (119°65′E, 29°08′N). The BPH was maintained on 50-day-old TN1 rice plants, while the *N. muiri* was maintained on *L. sayanuka* seedlings in separate cages (90 cm × 80 cm × 80 cm) for four generations in an insectary (27 ± 0.5 °C, 70–90% RH, 12D:12L). The parasitoid colony of *A. nilaparvatae* was established from seedlings of rice and *L. sayanuka*, which contained planthopper eggs parasitized by *A. nilaparvatae. A. nilaparvatae* populations were reared with BPH eggs in TN1 rice plants or reared *N. muiri* eggs in *L. sayanuka* plants for four generations in an insectary (27 ± 0.5 °C, 70–90% RH and 12D:12L).

### Population trend index of BPH and *N. muiri* on different host plants

We used an index (I) developed by ref. 32 to evaluate the population growth trends of *N. muiri* and BPH on *L. sayanuka* and rice plants, respectively. Each pot of TN1 and *L. sayanuka* was covered by netted cage and inoculated with a pair of BPH or *N. muiri* adults emerged in 4 hours, with 20 replicates for a planthopper specie in each host plant. The number of survival adults was observed daily. After egg hatching, the nymphs were counted and then removed every two days. When no more newly hatched nymphs were recorded from tested plants for continuous 4 days, the plants were cut and dissected under a dissecting microscope to record the unhatched eggs. The longevity of female and male adults, total fecundity (the sum of hatched nymphs and unhatched eggs), and egg hatching rate were calculated.

Newly hatched nymphs were individually reared on their parents’ host plats for further observation. Each potted plant covered by netted cage was inoculated with 10 new hatched nymphs. The survival and emergence of nymphs were recorded daily. Treatments were replicated 15 times and host plants were replaced or supplied with new ones in every 3 days. From the first day of adult emergence, the number and gender of emerged insects were recorded daily until all emerged. Afterwards, the emergence rate and sex rate were calculated.

### Parasitism by *A. nilaparvatae* to eggs of both BPH and *N. muiri* derived from different host plants

At the tiller stage, healthy potted TN1 rice plants or *L. sayanuka* plants were selected and stripped of old leaves, and then covered with an inverted transparent disposable plastic cup (diameter 7 cm, height 9 cm), which had a hole (diameter 2 cm) on the cup botton for plants growing. Five gravid female adults of BPH or *N. muiri*, respectively, were introduced into each pot and were removed after 48 hours. A pair of the newly (<4 h) adults of *A. Nilaparvatae*, emerged either from TN1 or *L. sayanuk*a was introduced into each cup. During the experiment, a 10% V/V honey water solution soaked cotton wool swab was placed in every pot as the food source. Parasitoids were removed after 24 h and the number of planthopper eggs, both parasitized and non-parasitized, were counted using a dissecting microscope after 4 days. Each treatment was replicated 30 times and arranged in a complete randomized design in an artificial climate chamber (27.0 ± 0.5 °C, 70–90% RH and 12L:12D).

### Functional responses of *A. nilaparvatae* on eggs from two hosts (BPH or *N. muiri*) reared by different host plants

Seedlings used for the functional response experiments were described above. TN1 and *L. sayanuka* potted plants were covered with mylar cages and inoculated with female adults of BPH and *N. muiri*, respectively at different densities (1, 2, 4, 6, 8 and 10 adults per pot) for 48 hours to provide varying eggs densities. A pair of freshly emerged of *A. nilaparvatae* parasiotids was introduced into each cage for 24 hours. The plants in each cage were dissected after 4 days under a dissecting microscope to record the number of healthy and parasitized eggs. During the study, a 10% V/V honey water solution soaked cotton wool swab was placed in every pot as the food source. Treatments were replicated 20 times and conducted in a climate chamber set at 27.0 ± 0.5 °C, 70–90% RH, and 12D:12L.

### Oviposition preference of *A. nilaparvatae*

TN1 and *L. sayanuka* seedlings were planted into the same pot and trimmed one tiller in rice and three tillers in *L. sayanuka* to maintain similar biomasses of the two plants in each experiment. One rice plant infested by BPH eggs and another plant infested by *N. muiri* eggs, respectively, were prepared to provide a range of prey egg densities. A pair of freshly emerged of *A. nilaparvatae* (1 d mated) was introduced into each pot for 24 h. During the study, a 10% V/V honey water solution soaked cotton wool swab was placed in every pot as the food source. The TN1 and *L. sayanuka* plants were dissected under a binocular microscope after 4 days to record the number of healthy and parasitized eggs. Treatments were replicated 20 times and conducted in the climate chamber set at 27.0 ± 0.5 °C, 70–90% RH, and 12D:12L.

### Field evaluation on the potential of *L. sayanuka* as a banker plant

A field experiment was conducted at the Jinhua experimental station (119°65′E, 29°08′N) of Zhejiang in China, in which we examined rice yields from replicated paddy field plots (20 m in length and spaced at 20 m intervals) under the following three treatments:

- *L. sayanuka* was planted in a 10 cm wide belt on the edge of paddy fields
- *L. sayanuka* was planted in a 50 cm wide belt on the edge of paddy fields
- Control – without *L. sayanuka* and other weeds on the edges of paddy fields

No pesticides were sprayed on paddy field plots, and the three treatments were replicated three times in a complete random split plot. *L. sayanuka* plants were introduced into field based on the experiment design prior to rice (Variety: Yongyou 12) transplanted. After 15 days, 100 female *N. miuri* per square meter were introduced to *L. sayanuka* seedlings, and the BPH population in rice field was assessed using the white enamel plate monthly internals. Five samples were collected respectively at 5, 10 and 15 m away from the bunds and the average population densities were compared among three treatments at the different distances, respectively.

### Statistics and analysis

The functional response data for *A. nilaparvatae* were analyzed using the Michael Marquardt method and the parasitoid model of Rogers^45^ and Royama^46^:


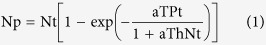


Where Np = the number of plants parasitized by each female wasp per day, Nt = the host density, a = the search effect (instantaneous attack rate), T = the total search time (one day), P = the parasitoid density, and Th = the treatment time.

The oviposition selectivity index for *A. nilaparvatae* on different hosts was calculated using the equation proposed by Manly *et al*.^47^:


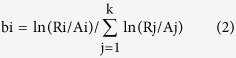


Where b_i_ is the probability of parasitism on host type i when all hosts provided could be used, R_i_ is the number of host type i without parasitized at the end of the experiment, Ai is the total number of host type i in the experiment, k is the number of host types (k = 2 in experiment 1. 2. 3).

The population trend index (I) for the two species of rice planthoppers were calculated according to the method of Qiao *et al*.^32^:


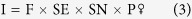


Where F is the fecundity of each female adult, SE is the hatching rate, SN is the emergence rate (nymph survival rate), and P_♀_ is sex ratio of the females.

All statistical tests were performed using SPSS v17. The significance of difference in the related parameters of population trend index of BPH and *N. muiri* was analyzed using the t-test. The percentage was first normalized by the arcsine square root transformation before analysis. The amounts of oviposition of both BPH and *N. muiri* on rice and *L. sayanuka* plants, and the oviposition preference of *A. nilaparvatae* to the eggs on different host plant were subjected to χ^2^ testing. One-way ANOVA was used to test for treatment effects of *L. sayanuka* on the population size of rice planthoppers and the Tukey post hoc test applied.

## Additional Information

**How to cite this article**: Zheng, X. *et al*. Use of banker plant system for sustainable management of the most important insect pest in rice fields in China. *Sci. Rep.* **7**, 45581; doi: 10.1038/srep45581 (2017).

**Publisher's note:** Springer Nature remains neutral with regard to jurisdictional claims in published maps and institutional affiliations.

## Figures and Tables

**Table 1:** Research that have implemented banker plant systems in greenhouse and outdoor vegetable and food crops.

Crop	Banker plant	Alternative host	Natural enemies	Target pests	References
**C**ucumber	Wheat, Barley	*Rhopalosiphum padi*	*Aphidius colemani, Aphidoletes aphidimyza*	*Aphis gossypii*	Bennison and Corless, 1993
Melon	Barley	*Schizaphis graminum*	*A. colemani, A. aphidimyza*	*A. gossypii*	Kim and Kim, 2004
Pepper	Broad beans	*Megoura viciae*	*A. aphidimyza*	*Myzus persicae*	Hansen, 1983
Cauliflower	Cabbage, turnip	*Brevicoryne brassicae, M. persicae, Macrosiphum euphorbiae*	*Diaeretiella rapae*	*Brevicoryne brassicae*	Freuler *et al*., 2003
Tomato	Papaya	*Trialeurodes variabilis*	*Encarsia Sophia*	*Bemisia tabaci*	Xiao *et al*., 2011
Vegetable	Ornamental pepper		*Amblyseius swirskii*	*B. tabaci, Frankliniella occidentalis, Scirtothrips dorsalis*	Xiao *et al*., 2012
Cangreen bean, Dusky bean	Corn	*Oligonychus pratensis*	*Feltiella acarisuga*	*Tetranychus urticae*	Xiao *et al*., 2012, 2013
Apple	Elder shrubs	*Aphis sambuc*	syrphids (Diptera: Syrphidae)	*Dysaphis plantaginea*	Brisbosia, 2003
Rice	*Zizania caduciflora*	*Saccharosydne procerus*	*Anagrus nilaparvatae*	*Nilaparvata lugens*	Yu *et al*., 1999; Yu, 2001
Rice	barnyard grass, *Echinochloa utilis*	*Sogatella uibix*	mirid bug, *Cyrtorhinus lividipennis*	*Sogatella furcifera, Nilaparvata lugens*	Matsumura and Urano, 2001

**Table 2:** Parameter estimates of the functional response of *A. nilaparvatae* reared from different host plants on the eggs of *N. lugens* and *N. muiri.*

Parasitoids	Hosts (eggs)	Instantaneous attack rate (a)	Processing time (Th)	*R*^*2*^	*P*	Maximum parasitized eggs (per day) (1/Th)
*A. nilaparvatae* emerged from *N. lugens* egg	*N. lugens*	0.782 ± 0.348	0.034 ± 0.005	0.3004	<0.001	29.41
	*N. muiri*	0.339 ± 0.150	0.037 ± 0.008	0.2638	<0.001	27.03
*A. nilaparvatae* emerged from *N. muiri* egg	*N. lugens*	0.365 ± 0.174	0.046 ± 0.008	0.1910	<0.001	21.74

**Table 3:** Preference test of *A. nilaparvatae* from different host plants on the eggs of *N. lugens* and *N. muiri* and Chi-square test for the hypothesis of no preference.

Parameter	*A. nilaparvatae* emerged from *N. lugens* egg	*A. nilaparvatae* emerged from *N. muiri* egg
Number of replications in each preference test	37	49
α	0.7635 ± 0.0493	0.5381 ± 0.0495
χ^2^	6.12 > χ^2^_0.05_	1.38 < χ^2^_0.05_

Degree of freedom = 1, χ^2^_0.05_ = 3.84 χ^2^_0.01_ = 6.64.

**Table 4:** Field evaluation of LNA banker plant system for rice planthoppers management (per hill).

Treatments	Jul 9	Jul 23	Aug 6	Aug 20	Sept 3
Control	9.1 ± 0.9bc	14.3 ± 1.4a	21.9 ± 2.0a	23.2 ± 2.1a	17.7 ± 1.9a
10 cm stripe	15.2 ± 1.7a	13.1 ± 1.1a	9.5 ± 1.0b	6.6 ± 0.8b	3.5 ± 0.6b
50 cm stripe	12.6 ± 1.4ab	12.3 ± 1.1a	7.1 ± 0.8b	5.7 ± 1.0b	2.4 ± 0.6b
F	4.988	0.728	34.862	49.862	48.401
*p*	0.011	0.489	<0.001	<0.001	<0.001

Values are mean ± SE. Means within a column followed by differing letters are differ significantly at *P* < 0.05.

**Table 5:** Parameter and population trend index of *N. lugens* and *N. muiri* on different host plants.

Host plants	Fecundity (per female)		Hatching rate (%)		Emergence rate (%)		Sex ratio (%)		Population trend index	
	*N. lugens*	*N. muiri*	*N. lugens*	*N. muiri*	*N. lugens*	*N. muiri*	*N. lugens*	*N. muiri*	*N. lugens*	*N. muiri*
Rice	476.3 ± 28.4a (20)*	3.6 ± 1.0b (20)	95.3 ± 3.5 (20)	23.3 ± 6.6b (7)	88.9 ± 1.2 (15)	32.5 ± 3.0b (5)	51.6 ± 3.9 (15)	44.0 ± 4.2 (3)	208.2	0.12
*L. sayanuka*	0b (20)	316.8 ± 24.9a (20)	—	91.2 ± 1.5a (20)	—	59.7 ± 3.6a (15)	—	53.6 ± 1.3 (5)	0	92.45
*t*	12.435	−9.488		−14.629		−4.333		−2.538		
*P*	<0.001	<0.001		<0.001		0.002		0.044		

Values are mean ± SE. Means within a column followed by differing letters are differ significantly at *P* < 0.05. *Parenthetical numbers in the first two rows indicate the number of concurrent replicates.
